# Were Rivers Flowing across the Sahara During the Last Interglacial? Implications for Human Migration through Africa

**DOI:** 10.1371/journal.pone.0074834

**Published:** 2013-09-11

**Authors:** Tom J. Coulthard, Jorge A. Ramirez, Nick Barton, Mike Rogerson, Tim Brücher

**Affiliations:** 1 Department of Geography, Environment and Earth Sciences, University of Hull, Hull United Kingdom; 2 Institute of Archaeology, University of Oxford, Oxford, United Kingdom; 3 Max-Planck-Institut für Meteorologie, Hamburg, Germany; New York State Museum, United States of America

## Abstract

Human migration north through Africa is contentious. This paper uses a novel palaeohydrological and hydraulic modelling approach to test the hypothesis that under wetter climates c.100,000 years ago major river systems ran north across the Sahara to the Mediterranean, creating viable migration routes. We confirm that three of these now buried palaeo river systems could have been active at the key time of human migration across the Sahara. Unexpectedly, it is the most western of these three rivers, the Irharhar river, that represents the most likely route for human migration. The Irharhar river flows directly south to north, uniquely linking the mountain areas experiencing monsoon climates at these times to temperate Mediterranean environments where food and resources would have been abundant. The findings have major implications for our understanding of how humans migrated north through Africa, for the first time providing a quantitative perspective on the probabilities that these routes were viable for human habitation at these times.

## Introduction

The role of the Sahara as a geographical filter and launch zone for dispersals of *Homo sapiens* out of Africa is a controversial topic [1], [2], [3], [4]. At issue is the observation that 130-100,000 years ago there was a marked increase in humidity in the present desert and adjacent regions [5], [6], [7], [8], which coincided with some of the earliest appearances of *H. sapiens* in both the Sahara and the Levant [9], [10], [11], [12], [13], [14], [15], [16]. During MIS 5 (Marine Isotope Stage 5) [17], high insolation in the northern hemisphere caused the African monsoon to assume a position up to 1,000 km north of its location today [18], [19], [20], [21]. Isotopic and geomorphic evidence suggests that rain falling on the north of the Trans-Saharan mountains then flowed towards the Mediterranean [2], [22], [23], potentially creating migration pathways via a series of ‘green corridors’ [4] and ‘mega lake corridors’ [24] across the Sahara. Dating of human fossils from the Levantine sites of Skhul and Qafzeh imply that early dispersals occurred along the eastern margins of the Sahara prior to ∼100 ka [13], [14], [15], while other craniodental remains show that populations closely resembling those of the Near East were simultaneously present in north western Africa [9], [16], [18]. Given the combined dating uncertainties, many of these fossils and associated archaeological Middle Palaeolithic/Middle Stone Age finds are likely contemporary with the last interglacial period of peak humidity attested in marine cores [20], [23] and stalagmites [6], [25].

However, supporting evidence for the ‘green corridor’ hypothesis remains subjective. Aside from topographic analysis, there has been no quantitative hydrological assessment (i.e. calculation of fluxes and balances of water) that could test whether these freshwater pathways across the Sahara were physically possible during the Eemian (MIS 5e). Surface evidence of fossil river systems and dated lacustrine records show there has been water in the region, but this does not provide an effective quantitative view of *when*, *where,* or *how much* water was present in the wider landscape. Interpretation of the archaeological record is equally subjective, as although the general direction of dispersal (northward from sub-Saharan Africa) and its general timing (last interglacial) are clear, these data remain ambiguous between a single trans-Saharan migration with delayed subsequent expansion, multiple migrations via a single route or multiple migrations via multiple routes. The existing evidence is not sufficient to conclude whether contiguous ‘Green Corridors’ existed at the right time for migration.

In this paper, for the first time, we simulate the balance and fluxes of water across this region. We use simulated precipitation from a state of the art Earth System Model (ESM) simulation of the Eemian (MIS 5e) climate to drive a combined hydrological and hydraulic model to reconstruct past rivers and flood events across 12,000,000 km^2^ of North Africa. For the first time, this reveals the *seasonal and spatial patterns* of Saharan surface palaeohydrology, predicting the presence of distinct river corridors and wetlands [24], [26]. Our simulations were carried out with the sole aim of testing the Green Corridor *hypothesis*; were contiguous corridors following surface water (i.e. rivers) really feasible during MIS 5e? Do all the buried rivers show the same history, or are there spatiotemporal differences? We achieve this by calculating a probability of surface water routes across the Sahara as a basis for further investigation.

## Methods

**Figure 1:**
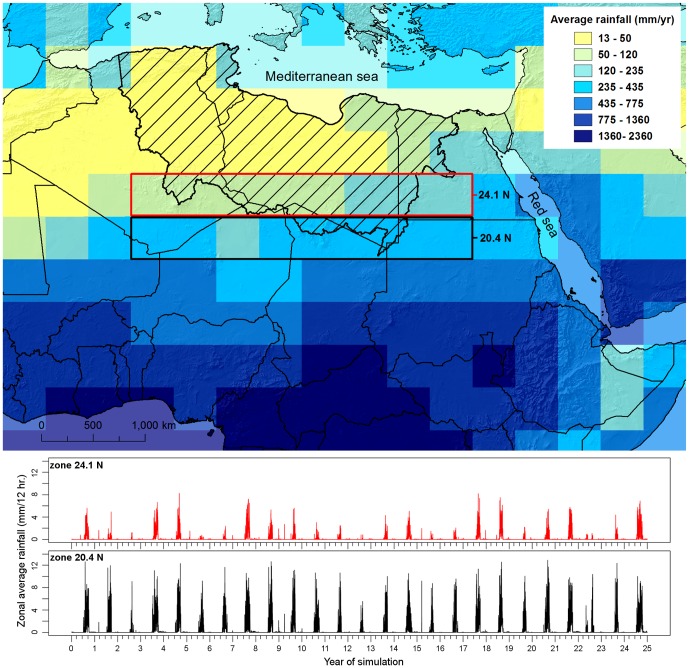
Palaeorainfall used to drive the combined numerical model. Yearly average rainfall from a 25 year snapshot of an ESM experiment and catchment area (hatched region) as well as the time series of zonally averaged precipitation for two stripes south of the catchment highlighting the (i) South-North gradient of rainfall during the wet seasons (June to September) and (ii) the modelled year to year variability of the monsoon system. The data is drawn from 12 hourly precipitation data produced during a time-slice experiment [27] of the last Interglacial (MIS 5e, ∼124 ka BP) performed by the fully coupled atmosphere-ocean- sea ice-biosphere general circulation model of the Max-Planck-Institute for Meteorology. The ESM consists of the spectral atmosphere model ECHAM5 [43] including the land surface model JSBACH [44] and a dynamic vegetation module [65] coupled to the general circulation ocean model MPIOM [45]. The model runs for the atmosphere at a truncation T31, which corresponds to a horizontal resolution of ∼300 km in the area under investigation.

Rainfall data of a palaeoclimatic simulation (124–125 ka) are taken from an experiment with the coupled atmosphere-ocean-sea ice-biosphere general circulation model (ECHAM5/JSBACH/MPI-OM) [27].This simulation provides a series of realistic scenarios in which the African monsoon sits c.700 km further north than at present [20] (see materials and methods section ‘Generating precipitation data’). The good performance of the ECHAM5 model is shown by its predictions for pre-industrial precipitation that can be compared to gauged data (see materials and methods section ‘Validation and Calibration’). For the period 125–124 ka BP a 25 year snapshot is taken with a sequence of 12 hourly, gridded data (3.75^o^ x 3.75^o^) across North Africa (Fig. 1). The northern edge of the MIS 5e monsoon spans the Ahaggar and Tibesti Mountains that are major topographic features covering a linear distance of ∼2500 km and including Southern Algeria, Southern Libya and Northern Chad with a maximum elevation of 2500 m. Previous spatial analysis of the regional topography [2] has shown there are major watersheds that are dry today but which would drain north from these mountains towards the Mediterranean. Satellite imagery reveals traces of major river channels linked to these watersheds, now partially buried under sand dune deposits [2], [22], [28]. Using a 1 km resolution digital elevation model (DEM) based on resampled GMTED 2010 topographic data (vertical accuracy RMSE 26–30 m) [29] the watersheds draining North from Ahaggar and Tibesti were delineated (see materials and methods section ‘Issues with the DEM’), excluding areas draining into the Nile basin to the East and the Senegal and Niger basins to the west. This area is shown as the shaded area in Figure 1 and is the focus of this study. The DEM is therefore based upon the present day topography though as sea level was up to 20 m higher during the earliest millennia of MIS 5e when our experiments are set [30], we adjusted the DEM by subtracting 20 m from all elevations, and redefined the coastline along newly submerged locations.

Simulated rainfall data at 12 hour resolution were fed into a hydrological model [31] for each climatic grid cell which generated surface runoff across the 1 km DEM at 1 min resolution. Surface runoff was routed across this DEM using a 2d hydrodynamic flow model based on the Lisflood-acc model [32], (see material and methods section ‘Hydraulic Model’). As our aim was to test the viability of fluvial corridors, and for model parsimony, groundwater recharge was not simulated, though we acknowledge that there is evidence for palaeolakes in the region that were ground water fed [33] and these lakes could have formed part of a migratory route [2], [26]. A series of 25 year simulations were carried out with high, medium and low infiltration/evaporation rates (1.5, 3 and 6 m yr^−1^) [34], [35], [36] and for every model time step water was also removed from grid cells to account for evaporation and infiltration. Hydrological model outputs were plotted as probabilities of surface water being present at a location varying between 0 and 1. These were determined by recording daily outputs of water depths and summing over a 25 year simulation period the number of days where water depths greater than 0.1 m were present. The use of 1 km grid cells imposes limitations as all river channels must be a minimum of 1 km wide resulting in excessive channel width/depth ratios under lower flow conditions. This, along with no groundwater recharge, leads to an underestimation of cell flow depths and thus a poorer drainage network connectivity/delineation as well as an over estimate of water loss from infiltration/evaporation due to too large water surface/channel bed areas. This conservative model configuration gives us a far higher confidence in our results as it reduces the possibility of overestimating inundation timing and extents. Additional shorter runs were used to test model sensitivity to altering hydrology and rainfall rates and to demonstrate the model’s validity. Further validation of the palaeohydrology modelling method is provided in materials and methods section “Validation and Calibration”.

## Results

**Figure 2:**
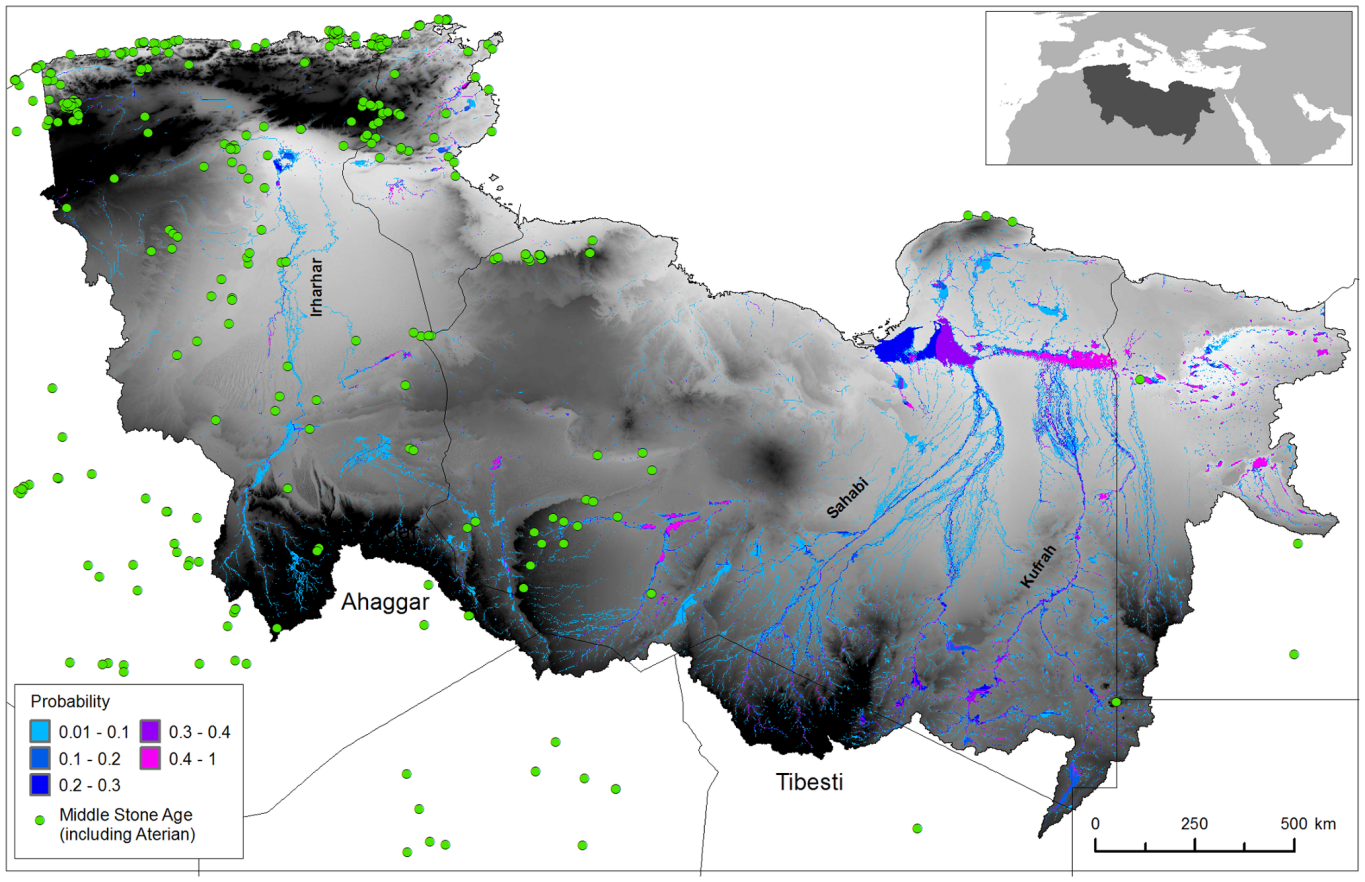
Simulated probability of surface water during the last interglacial. This figure details Archaeological sites, and an annual probability that a location has surface water. The archaeological data are derived from a number of sources (including [42], [66], [67], [68]. The findspots are characterised by Aterian and Middle Stone Age artefacts such as bifacial foliates and stemmed Aterian points and/or typical ‘Mousterian’ points, side scrapers and Levallois technology. Most are represented by surface scatters but where stratified examples exist these can be shown by dating (OSL and U-series techniques) and geomorphological setting to belong within MIS 5e [41], [42].

**Figure 3:**
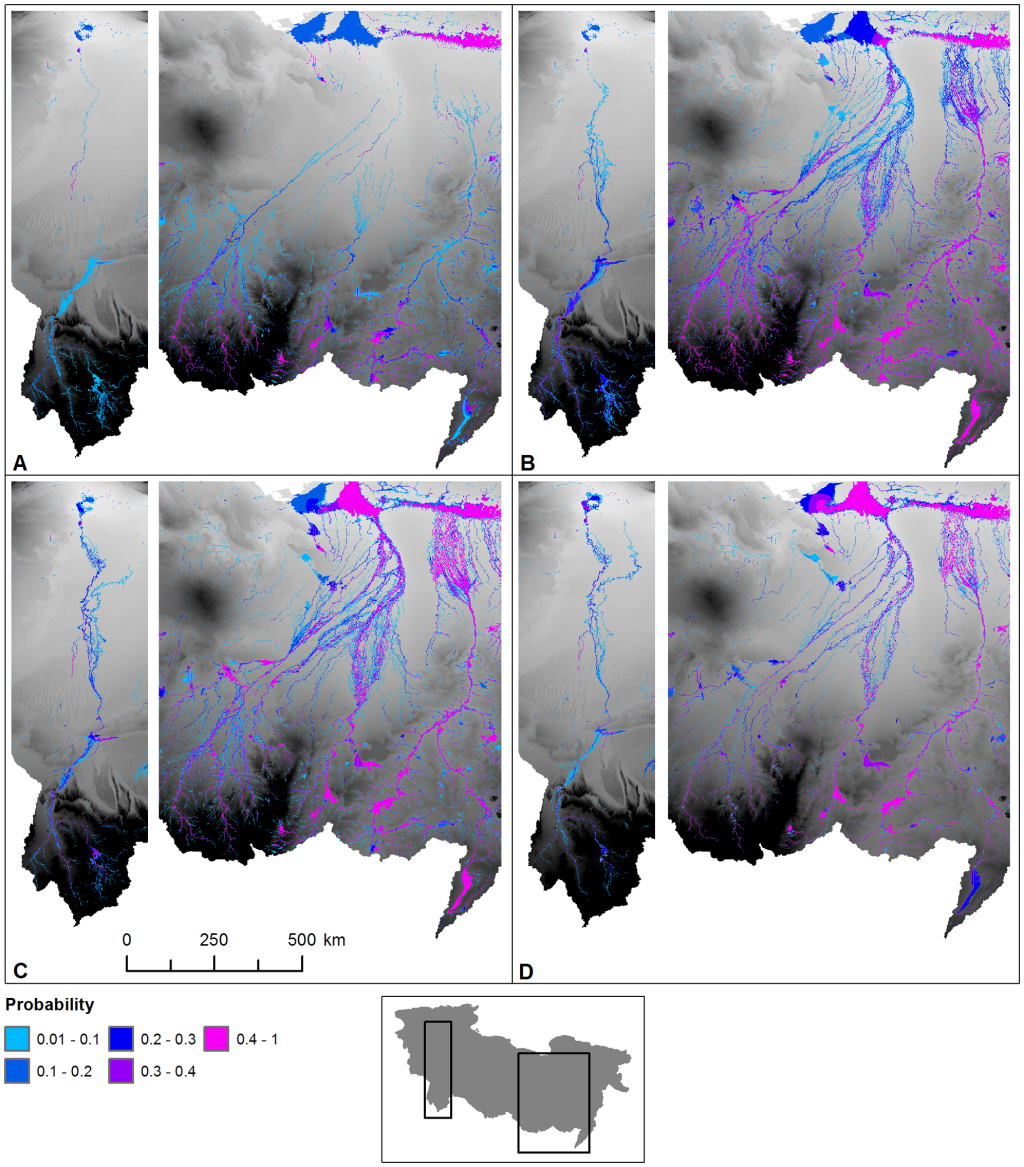
Seasonal flow of surface water across the Sahara during the last interglacial. Monthly probability of surface water being present for (a) August, (b) September, (c) October and (d) November. These illustrate the hydrodynamics of the system simulated by the large scale 2d hydraulic model that routes a flood wave of water north across the desert from the mountains.

Figure 2 demonstrates the existence of a series of extensive ephemeral and perennial river systems draining North from the Ahaggar and Tibesti mountains across the Sahara to the Mediterranean during the period 125–124 ka BP. Some channels dissipate in the desert, but some converge forming three main systems; in the West the Irharhar river draining into the Chott Melrir basin, and to the East two larger systems named the Sahabi and the Kufrah (Fig. 2). As the rainfall is associated with the monsoon, flow is highly seasonal and the Irharhar river is ephemeral, flowing for ∼3 months. The Sahabi and Kufrah systems are close to perennial, due to larger contributing areas in the catchment headwaters located in the monsoon belt (Fig. 1). Figure 3 demonstrates the seasonality of flow in all three systems with precipitation in August taking over two months to reach the coast or near-coastal lake systems. The distinct climatic zoning (Fig. 1) means all three rivers are allogenic, losing water along their length with little or no hydrological contribution once they leave the mountains/uplands. In addition to rivers, the simulations predict massive lagoons and wetlands in NE Libya some of which are extensive (>70,000 km^2^). These are also fed from the Jebel Akhdar in Cyrenaica, which also received higher rainfall during this period. There are smaller lakes forming in Tunisia and Algeria due to water supplied via the Irharhar river system.

Figure 2 and 3 present results from the conservative medium evaporation/infiltration scenarios, and are based on all 25 years of simulated hydrology - as rainfall within the ESM varies year to year. Simulations with higher evaporation/infiltration rates (6 m yr^−1^) gave the same drainage network but with lower probabilities. Simulations giving rise to wetter (1.5 m yr ^−1^ evaporation/infiltration) conditions increased the longevity of a channel’s wetness but did not introduce any new major water courses, nor significantly increase the wetted area or size of any lake systems.

## Discussion

This study provides the first strong quantitative evidence for the presence of three major river systems flowing across the Sahara during MIS 5e. We simulated three river systems that are now largely buried by dune systems, but when flowing would have provided fertile habitats for flora and fauna in proximity to the channels [22]. Notably, the Sahabi and Kufrah would be major river systems with monsoon discharges significantly in excess of 2500 m^3^ s^−1^ and an extensive system of anabranches and wetlands. In the Libyan Kalanschiou region, the green corridor would have been 100 km wide, substantial and largely perennial. This reconstruction is highly compatible with evidence of widespread palaeosols deposited on the margins of this system during the less pronounced Holocene humid period [22]. Here we have simulated one wet phase, but this research strongly supports the occurrence of similar ‘Green Saharas’ recorded in the marine [37] and terrestrial [26] archive.

Our simulation results quantitatively confirm previous hypotheses of these rivers shown in geomorphic and geochemical data [2], [4], [22], [23], [24]. For example, the radio-isotopic composition of the water identified in the Ionian Sea [4], [38] indicates that it was precipitated in the basaltic trans-Saharan range in Southern Libya as shown by our results. Runoff flowing rapidly to the coast, in a manner highly comparable to our simulated runoff waves, is shown by the light oxygen isotopic composition of the water flowing into the Mediterranean at this time [20]. Furthermore, the river systems that our research simulates are consistent with the well-preserved drainage network that has been identified in these regions by fieldwork and from satellite imagery [22], [24]. Overall, our confirmation that these hypotheses are physically realistic allows us to move on to questions of how and when the rivers operated, rather than their existence.

Whilst we cannot state for certain that humans migrated alongside these rivers, the shape of the drainage systems indicate that anyone moving from south to north from a 2000 km wide region in the mountains would be funnelled into three clear routes. There is also a clear geographical split, with a 2000 km gap between the destinations of Irharhar and the combined Sahabi and Kufrah systems.

Despite being ephemeral, the Irharhar river corridor could be the most suitable for dispersal of hominids beyond the Sahara. Uniquely, the Irharhar extends from *humid* to *humid* climes - ranging from the monsoonal Ahaggar and Tibesti region to the North Western Mediterranean climate zone that also received substantial winter rainfall (Fig. 1). High humidity in the destination region during the last interglacial is confirmed by the presence of significant water near the Chott Melrir basin [39]. Whilst the more extensive Sahabi and Kufrah also traverse the Sahara, their downstream limits remain within the arid/semi-arid regions [40].

Support for the significance of the Irharhar river corridor is provided by the high number of Middle Stone Age archaeological sites concentrated in the western region (Fig. 2). Many of these locations contain Levallois lithic artefacts with Aterian affinities that on comparative grounds can be plausibly dated to the last Interglacial [41], [42]. It is highly likely given the existing artefact distributions that humans migrated northwards from the relatively humid Trans-Saharan mountainous zones to the Maghrebian Mediterranean biome (Fig. 2). The loose clustering of sites along our simulated Irharhar river and associated channels implies this as a preferred route of dispersal. Furthermore, as the simulations are driven by present day topography, if the dune systems in this region were removed or reconfigured the Irharhar could flow further to the West. In contrast, the eastern region has a surprising lack of archaeological evidence despite the extensive simulated palaeo-river courses. It is likely that further surveys in this area will provide substantial evidence of Middle Stone Age activity, especially in the areas of buried palaeochannels. However, continued absence of this critical evidence of human migration would confirm our suggestion that a key factor in the western distribution of sites was the attractiveness of the richer Mediterranean-type environments of the Maghreb, which would have promoted permanent settlement in the region and further transit in both directions along the Irharhar river corridor.

## Conclusions

For the first time, our simulations demonstrate that Saharan “Humid Corridors” were highly likely during the last interglacial strongly re-affirming the viability of these routes as migratory corridors for early hominids. This research provides an unprecedented means of developing new hypotheses for past human, faunal and floral activity in this region and for validating the performance of palaeo climate simulations.

## Materials and Methods

### Overview of methodology for simulating surface palaeo hydrology in North Africa

**Figure 4:**
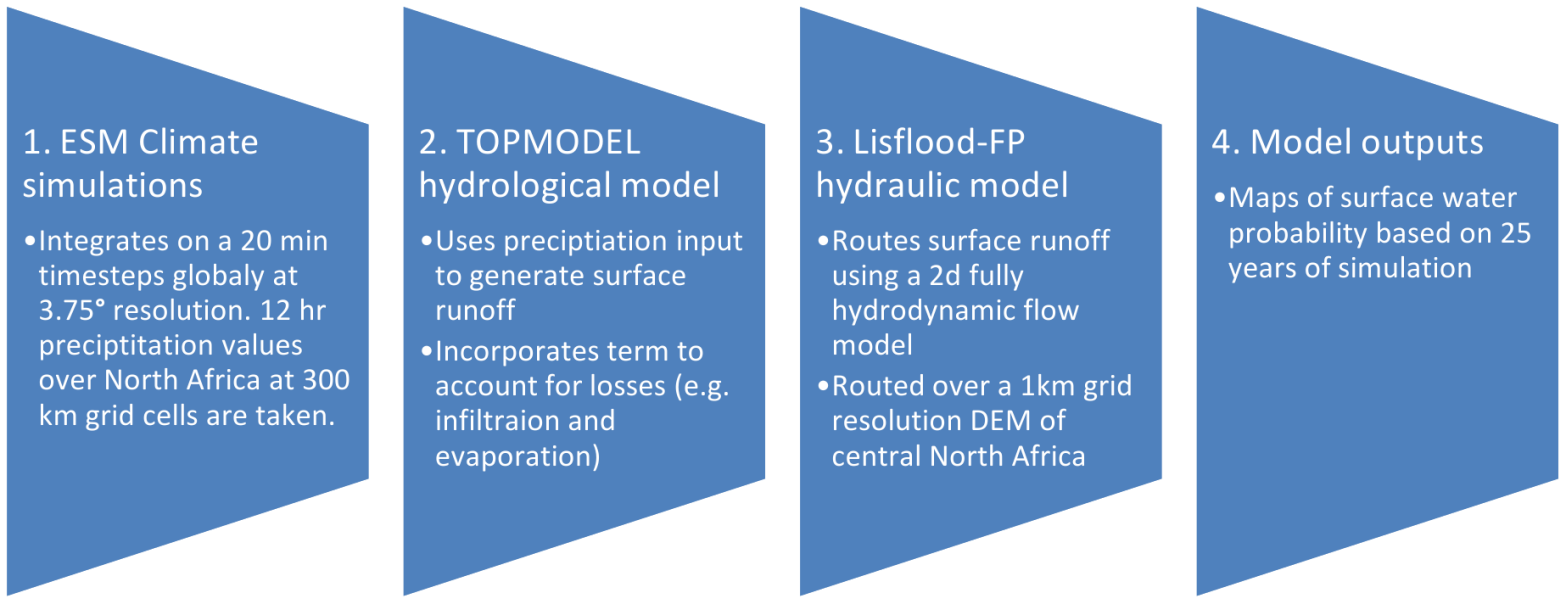
Combined model structure. Schematic of how the modelling sections combine to provide hydrological reconstructions of North Africa surface water.

This section describes the numerical modelling procedure and set up for simulating the palaeohydrology of North Africa. The methodology follows the structure outlined in Figure 4 with three numerical models working together: The Earth System Model (ESM) develops precipitation patterns that are then used as an input for catchment hydrological modelling and finally a hydrodynamic flow model to simulate patterns of surface inundation. Each section from this will be discussed in the following sections along with sections detailing calibration and testing of the model, and preparation of the DEM.

### Generating precipitation data from the ESM: Model configuration and experimental setup

The ESM used in this study is the Max Planck Institute coupled atmosphere/ocean general circulation model. It consists of the spectral atmospheric component ECHAM5 [43] running at truncation T31 (approximately 3.75**°**) and a vertical resolution of 19 levels up to 10 hPa. The underlying land component includes a dynamical land surface scheme (JSBACH, [44]) running on the same grid. The Ocean model MPI-OM [45] operates at resolution GR30 (app. 3**°**) with 40 vertical levels (30 levels within the top 2000 m). A dynamic-thermodynamic Hibler-type model simulates sea-ice [46]. The coupling between atmosphere and ocean is done by OASIS [47] on a daily basis without any flux correction [48] which leads to a more realistic heat transport in the ocean than flux-corrected models. Momentum, heat, and freshwater fluxes are transferred from the atmosphere to the ocean, while sea surface temperature, sea ice thickness and fraction, snow cover on sea ice, and surface velocities are returned from the ocean to the atmosphere. Freshwater fluxes from land to the ocean are calculated by a simple river run-off scheme [49], [50].

The integration time step of ECHAM5 at a resolution of T31 is 20 min, MPI-OM has a time step of 2.4h at GR30 resolution. For the atmospheric component of the ESM, six hourly values are written out during the simulation. 12 hr sums of precipitation (convective plus large scale precipitation and snow fall) are the underlying data used in our investigation.

Results out of two different transient experiments based on the same model version are used in this study: a transient simulation of MIS 5e (125 ka BP) and a transient simulation starting at 6 ka BP until pre industrial climate [27], [51]. Both simulations follow the same spin-up procedure to create starting conditions for the experiment. By integrating the control run (assumed to be in equilibrium with pre-industrial boundary conditions) for a further 2500 model years the orbital parameters are changed accordingly [52]. Our investigation is based on MIS 5e data after a further 1000 years of integration (125 ka BP conditions) and for preindustrial climate the last 50 years of the 6000 yr transient simulation are taken (0 ka BP). All experiments do not vary atmospheric greenhouse gases and are prescribed as constant (CO_2_ to 280 ppm, CH_4_ to 700 ppb, N_2_O to 265 ppb).

### Hydrological model: TOPMODEL

The precipitation output from the ESM (section above) is used to drive the hydrological model. This is a modification of TOPMODEL [31] and is used to generate a combined surface and sub-surface discharge (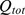 ) for all cells. This is calculated according to equation 1, where *T* is the time step (in seconds), *r* the rainfall rate (in m^−1^hr^−1^) and *m* is a parameter that controls the rise and fall of the soil moisture store (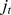 ) that is calculated from the value of 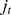 in the previous iteration (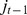 ). *m* can be derived from the recession curve of the flood hydrograph [53]. 
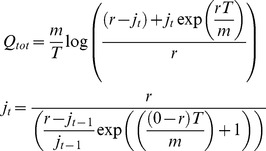
(1)


If there is no precipitation (*r* = 0) then equation 2 is used.
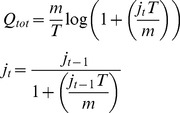
(2)


Equations 1 and 2 are calculated for each Digital Elevation Model (DEM) cell (1 km resolution) within the larger cells that contain the precipitation data from the ESM. This provides a 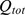 for every cell within the study area – that varies according to the precipitation amount falling within that cell. 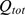 is then converted into a flow depth and then routed according to the hydraulic model outlined below.

### Hydraulic model: The LISFLOOD-FP flow model

LISFLOOD-FP is a one dimensional inertial model that is applied in the x and y directions to simulate two dimensional flow over a raster grid. The method is first order in space and explicit in time, but uses a semi implicit treatment for the friction term to aid stability (See [32]) To calculate the flow (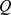) between cells equation 3 [32] is used:
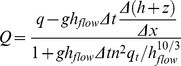
(3)


Here *q* is the flux between cells from the previous iteration (m^2^s^−1^), *g* is acceleration due to gravity (ms^−1^), *n* is Manning’s roughness coefficient (m^1/3^s^−1^), *h* is depth (m), *z* is elevation (m), 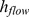 is the maximum depth of flow between cells, *x* is the grid cell width (m), *t* is time (s) and *i* and *j* are cell co-ordinates. Having established the discharge across all four boundaries of a cell, the cell water depth (*h*) is updated using equation 4:
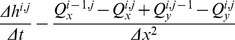
(4)


The final part of the LISFLOOD-FP formulation is the time step (t) that is controlled by the shallow water CFL condition as equation (3) is a true shallow water model:
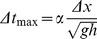
(5)


Where 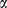 is a coefficient typically defined between 0.3 and 0.7 [32]. This term is required to maintain numerical stability in all circumstances. As equation 3 demonstrates this is strongly influenced by the grid cell size and the water depth.

LISFLOOD-FP has been extensively tested and compared to other flow models in benchmarking studies [54], and the stability of the original [32] numerical solution at low friction (n < 0.03) has been substantially improved by [32], [54], [55]. The new version of the model used in this analysis is capable of simulating flow depths and velocities within 10% of a range of industry full shallow water codes [54]. The advantage of using LISFLOOD-FP compared to an explicit full shallow water model is that it is considerably faster and consumes less computation power. Lisflood-FP has also been used to simulate inundation extent, water depth, and, wave propagation for very large rivers including the Amazon [56], Niger [57], and Ob [58]. In this study, the TOPMODEL hydrological and LISFLOOD-FP hydraulic model are combined within the CAESAR-Lisflood model framework [59].To account for water losses, water depths are subtracted from each grid cell every simulated day according to a combined evaporation/infiltration rate. This parameter is applied globally even though evaporation and in particular infiltration will vary spatially and across different regions modelled here. However, as this study aims to test a hypothesis rather than generate precise volumes of water delivered, we feel it is important to use (overly high) pessimistic evaporation/infiltration rates to avoid “positive errors” (e.g. river reaches coast) arising from application of an incorrect infiltration in a specific, remote and therefore poorly known location. In this way, we can be confident that surface water was likely to be at least as widespread as our model outputs show, and “false positive” responses from the model are suppressed.

### Validation and Calibration of surface palaeo hydrology models

Validating this modelling approach was important for two reasons; firstly, to show that the combined hydrological/hydraulic model was performing correctly; and secondly to evaluate the performance of the ESM by checking outputs from pre-industrial ESM simulations. This could be carried out by comparing simulated to measured river flow records on a catchment driven by measured (rain gauge) and simulated (ESM) precipitation data.

However, it was difficult to find a river system of suitable size and similar climate that contained a gauged record of both rainfall and discharge in order to validate and calibrate the model. We chose to validate and calibrate the combined models on the upper Nile before the construction of the main dams (1900–1920). For this period monthly rainfall gridded across the basin [60], and discharge data (at Aswan) were available [61]. The Nile has its hydrological idiosyncraticies – and despite most of its discharge originating from the Blue Nile, it still represents a very large seasonal river that flows across a substantial dry area involving significant water losses.

**Figure 5:**
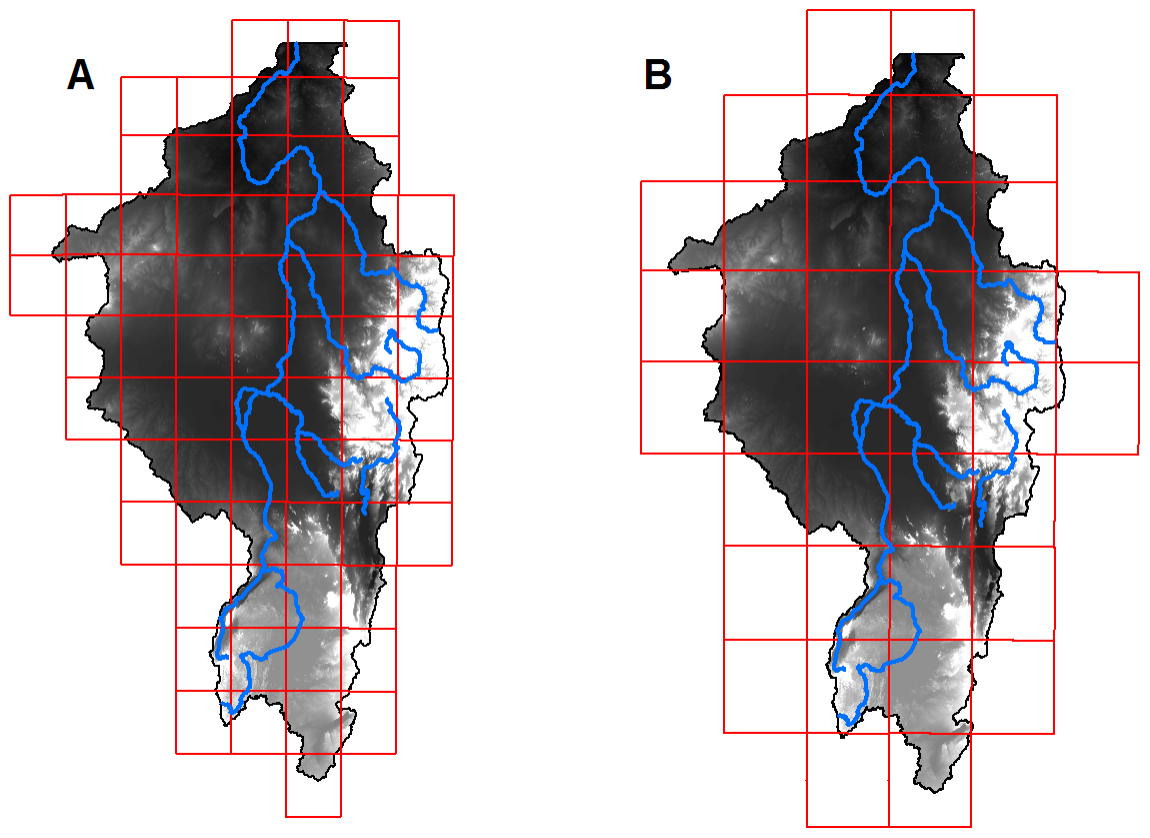
Gridding of observered and ESM simulated precipitation. DEM of the Nile, upstream from Aswan, showing grid of different observed rainfall values (a) and grid of pre-industrial ESM precipitation values (b).

**Figure 6:**
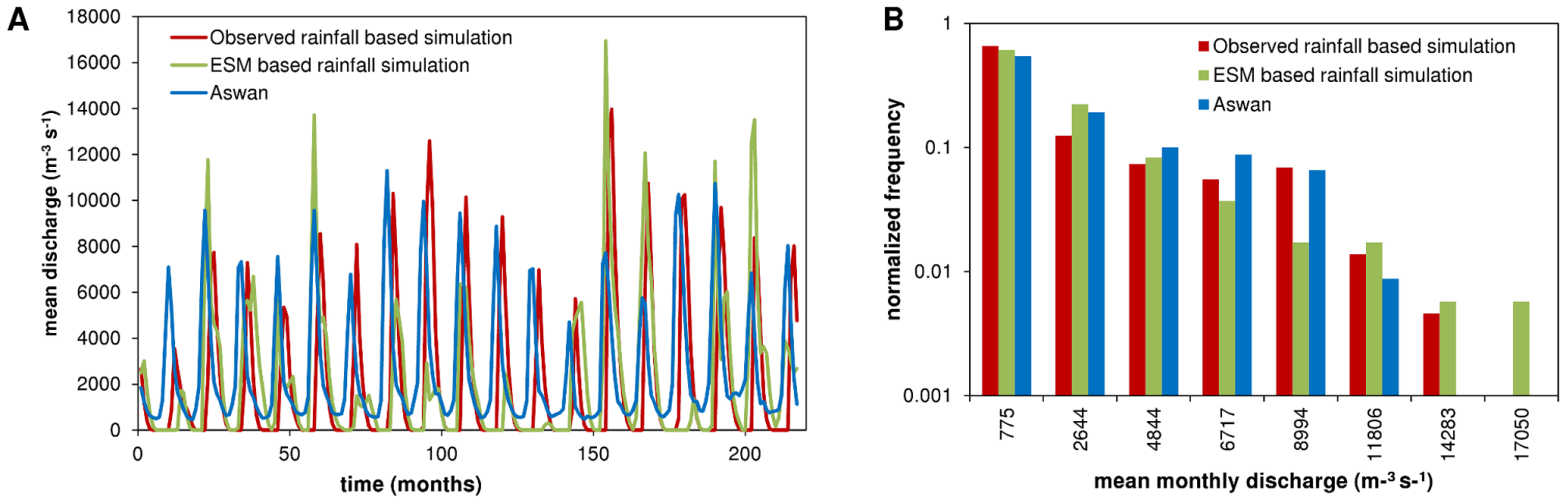
Observed vs simulated discharges at Aswan for the Nile validation. Mean monthly discharge for the upper Nile recorded at Aswan from 1901–1920, discharge produced from the combined models driven with observed gridded monthly land-surface precipitation totals from 1901–1920, and driven with ESM pre-industrial climate (a). Comparison of magnitude and frequency of mean monthly discharge at Aswan, discharge produced from combined model simulations driven with observed precipitation, and ESM pre-industrial climate (b).

A 1 km DEM was created by resampling and conditioning the GMTED 2010 DEM product (for more discussion see the material and methods section ‘issues with DEM’) and the measured monthly rainfall totals for the 68 grid cells were used as the input to the hydrological model (Figure 5a). Simulations were performed both using precipitation input from an instrumental (rain gauge) dataset (monthly totals) and also using the pre-industrial output of MPI-ESM (12 hour totals). Monthly discharges were then recorded and compared to the gauged record at Aswan. The evaporation/infiltration term (section 3 above) was used to calibrate model outputs and a value of 0.015 m day was found to give the best fit to the observed data. This is shown in Figure 6a by the red line, where blue corresponds to the gauged record. It is worth noting that some uncertainty surrounds the gauged record as it is based on release and fill records at the first Aswan dam [61].

**Figure 7:**
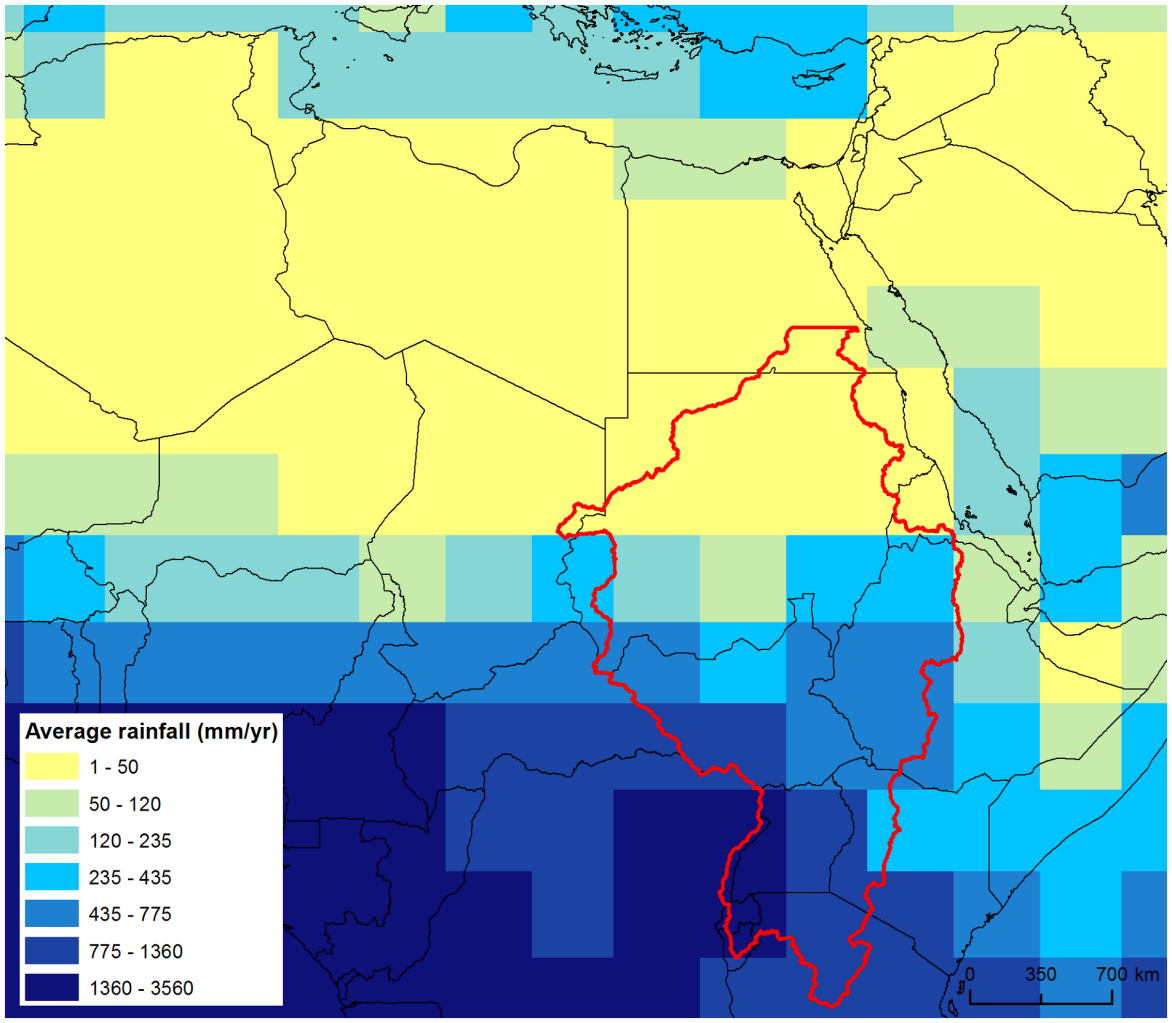
Pre-industrial precipitation patterns from the MPI-ESM. Yearly average rainfall from a 50 year snapshot of the MPI-ESM experiment of pre-industrial climate, (280 ppm CO_2_ out of a transient simulation).

**Figure 8:**
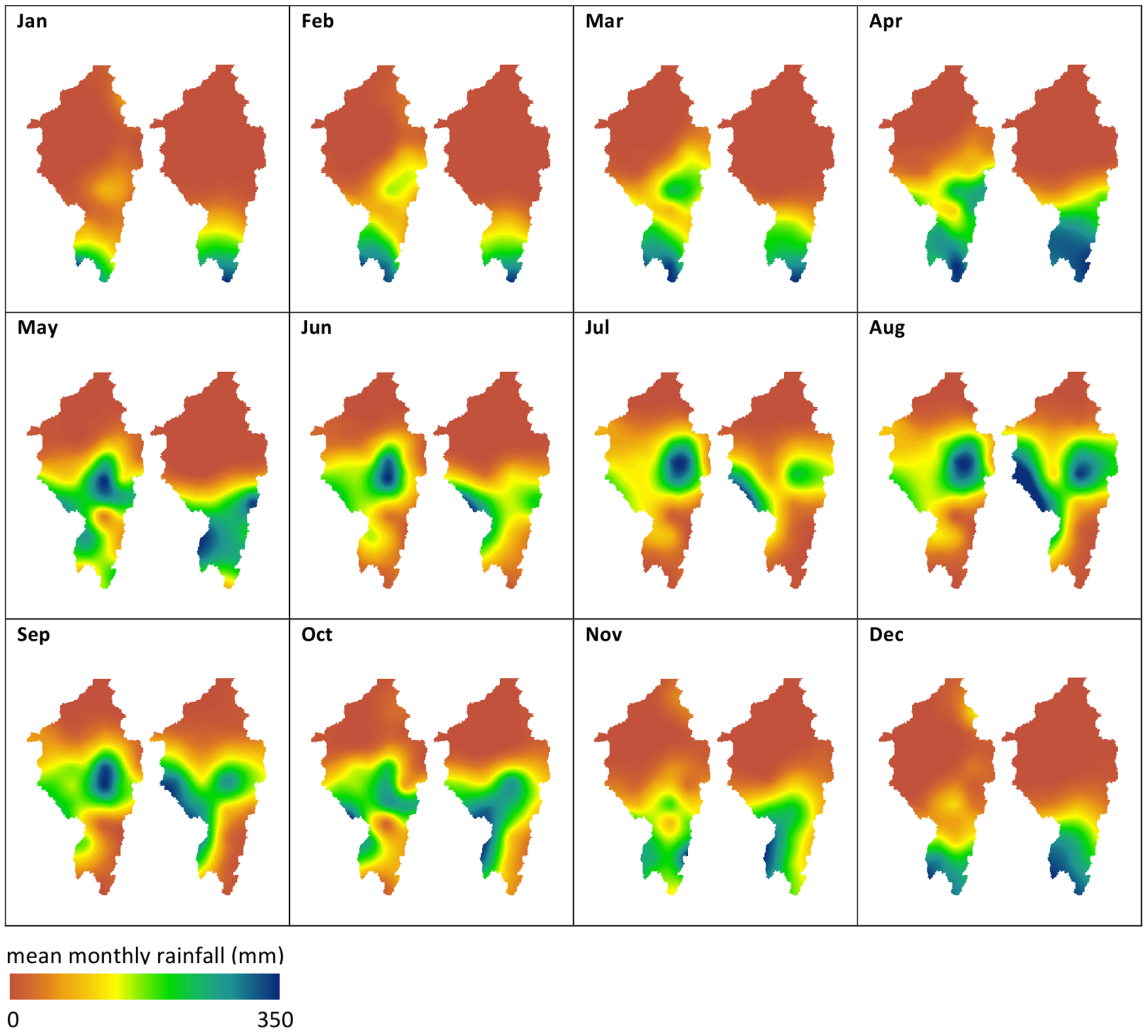
Comparison between observed and ESM simulated rainfall for pre-industrial scenario. Comparison between observed rainfall (maps on left) and pre-industrial ESM (maps on right). ESM cells overlaying the upper Nile catchment were selected and mean monthly totals were calculated for a 50 year time series. Mean monthly totals for observed rainfall cells were calculated for cells of the catchment from the time period 1901–1920. To resolve differences in spatial resolution between both datasets, (Figure 5a,b), the centroids of observed and ESM data were chosen to interpolate mean monthly rainfall using a spline technique, at 10,000 m resolution.

Using the calibrated evaporation/infiltration term, the hydrological/hydraulic model was run using rainfall data from the ESM for a pre-industrial scenario (Figure 7). Outputs from this simulation are shown by the green line in Figure 6a. Clearly we cannot expect as good a match as from the recorded rainfall data – as the ESM simulated rainfall will not have the same patterns of wetter or drier years etc. Therefore, to provide a more meaningful validation we have plotted a histogram of monthly discharges for both simulations and the observed data (Figure 6b). This shows an excellent match between the discharges recorded at Aswan and simulated discharges using observed rainfall (χ^2^ = 48, *p* = 1) and ESM rainfall (χ^2^ = 48, *p* = 0.06), giving us a high degree of confidence in the performance of both the ESM and the hydrological/hydraulic models. Further comparison between the ESM and observed rainfall record is shown in Figure 8. The ability of the ESM to predict pre-industrial distributions of precipitation is clearly demonstrated.

We emphasise that differences do occur under low flow scenarios between the simulated and observed data. This reflects the conservative nature of the model described above, which results in rivers that should have very low flow being represented as dry. From the perspective of freshwater routing across North Africa these differences are negligible; almost all of the freshwater transport occurs during the flood phase which we resolve extremely well. Resolution of this “low flow” issue would require simulation of groundwater recharge, and given that we already simulate the annual flux values very well this is considered beyond the scope of this study.

### Issues with the DEM

**Figure 9:**
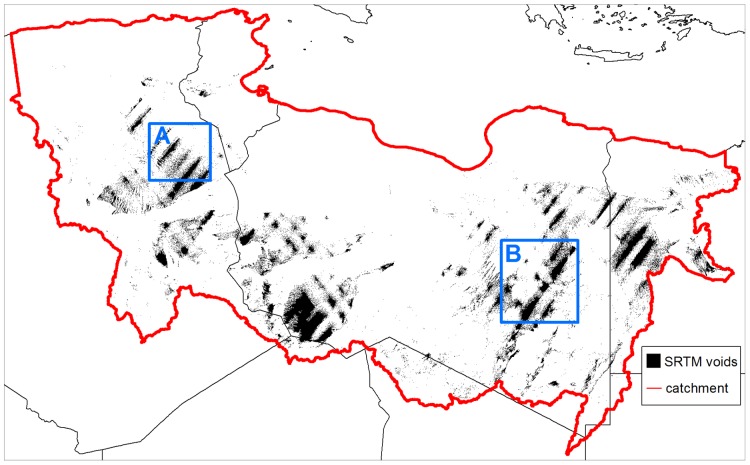
SRTM DEM data voids in the study area. Areas of STRM voids within the study area, showing insets used in Figure 10.

**Figure 10:**
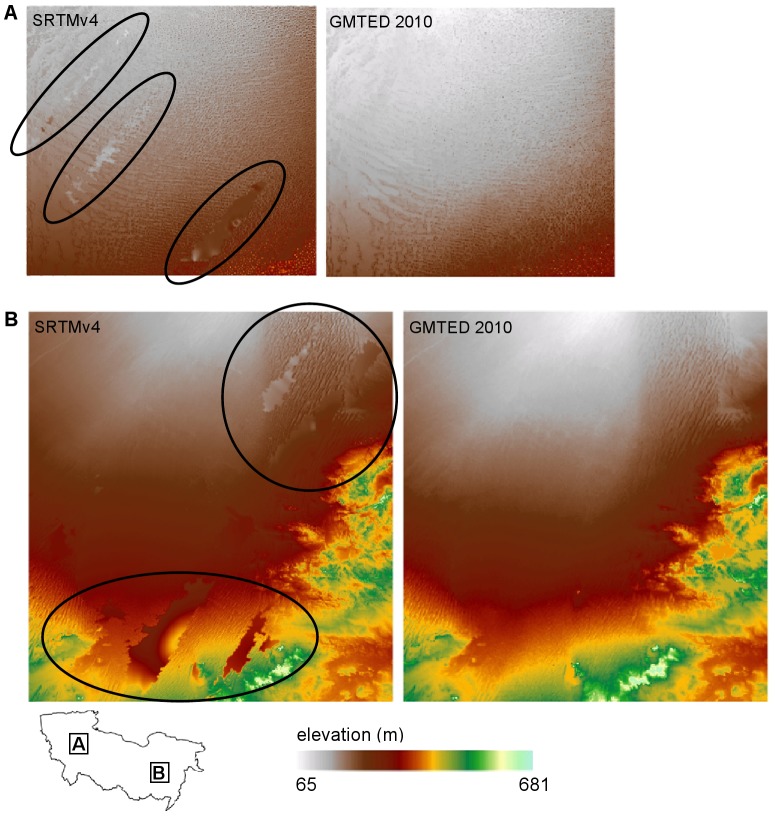
Erroneously filled voids in STRM data. Elevation values from void filled SRTM v4 and GMTED 2010 data. Examples of large errors in elevation values are circled.

To represent the surface within our model, we first considered the 90 m resolution SRTM v1 elevation data. However, the catchment modelled in this study has a far from complete coverage from the SRTM with 7% of the catchment area occupied by voids where no data were collected (Figure 9). Efforts have been made to fill these voids with auxiliary elevation data [62] to produce a finished elevation data product (SRTM v3 or v4) that is widely available for download. These SRTM v3/v4 data were used in previous studies delineating possible drainage patterns in North Africa [2], [26]. However, we chose not to use these data as the auxiliary data used to fill voids in SRTM v3/v4 for North Africa has considerable limitations. Post processed SRTM v3 or v4 data contains large vertical errors (+/–160 m) where voids have been filled [63], rendering it unacceptable for use within a flow model. This error is sufficiently pronounced to be apparent through a visual inspection of the SRTM v4 DEM, where patches of elevation discontinuities are apparent when compared to surrounding elevations (Figure 10). Clearly erroneous, such major discontinuities can have a major impact on the basic flow accumulation analysis used to delineate stream networks – let alone more sophisticated hydraulic modelling methods we use here.

For this reason, we decided that the SRTM v3 or v4 data were unsuitable as we required a representation of the topography that was not interrupted by erroneous void filling. An alternative to the SRTM v3 or v4 data is the Global Multi-resolution Terrain Elevation Data 2010, [29] that where available uses SRTM data, and augments this with SPOT 5 Reference3D elevations to fill voids in the SRTM. GMTED 2010 represents a significant improvement over previous efforts to fill voids in SRTM data because the vertical error of the SPOT 5 Reference3D data is +/–10 m [64] (an order of magnitude smaller than SRTM v3 or v4). The difference this makes is apparent by comparing the GMTED 2010 to the SRTM v4 in Figure 10. The huge artefacts in the DEM that are present in the SRTM v4, with a maximum area of 3300 km^2^, are not present in the GMTED 2010 data.

Therefore, for this study we used the GMTED 2010 (250 m spatial resolution) data that was re-sampled to 1000 m and stored as float elevation values. The resulting DEM was filled and large sinks representing natural depression were preserved in the DEM. (e.g. Qattara Depression). A drainage network was calculated using a D8 flow routing algorithm and a 1 m wide, 1 m deep drainage network was burned into the DEM, to reflect sub-grid scale river channel networks known to exist throughout the region [28]. To account for a higher sea level at 125 ka BP, we adjust the DEM by subtracting 20 m from all elevations, and redefined the coastline along newly submerged locations.
