# Correction: Were Rivers Flowing across the Sahara During the Last Interglacial? Implications for Human Migration through Africa

**DOI:** 10.1371/annotation/0a0303fa-ae35-4100-9f8d-c9ad65d49897

**Published:** 2013-10-22

**Authors:** Tom J. Coulthard, Jorge A. Ramirez, Nick Barton, Mike Rogerson, Tim Brücher

This article was republished in order to correct an error in the Editor name.

